# Understanding the Role of Polyols and Sugars in Reducing Aggregation in IgG_2_ and IgG_4_ Monoclonal Antibodies During Low-pH Viral Inactivation Step

**DOI:** 10.3390/ph18121846

**Published:** 2025-12-03

**Authors:** Shiqi Hong, Tao Peng, Lucas Yuan Hao Goh, Keat Theng Chow, Deepak Bahl, Vinod Tuliani

**Affiliations:** 1 Pharma Applied Sciences, Roquette Asia Pacific Pte Ltd., Singapore 138588, Singapore; 2 Roquette Pharmaceutical Innovation Center, Lower Gwynedd Township, PA 19002, USA

**Keywords:** low pH, virus inactivation, monoclonal antibodies, aggregate, polyols, sugars, conformational stability, colloidal stability

## Abstract

**Background/Objectives**: Low-pH treatment of monoclonal antibodies (mAbs) is commonly employed to inactivate enveloped viruses following Protein A capture chromatography. However, this treatment can sometimes induce conformational changes and aggregation in pH-sensitive proteins. This study investigates the stabilizing effects of four compendial excipients—mannitol, sorbitol, trehalose, and sucrose—on aggregation, as well as on the conformational and colloidal stability of pembrolizumab (IgG_4_) and denosumab (IgG_2_) in the context of low-pH viral inactivation (VI) operations. **Methods**: Following Protein A chromatography, pembrolizumab and denosumab were subjected to low-pH incubation and neutralization to mimic the conditions encountered in the VI step. Excipients (10% *w*/*v*) were added to the eluate, and aggregation was assessed post-neutralization. Effects on conformational stability (measured by thermal unfolding) and colloidal stability (via diffusion interaction parameter K_D_) were investigated. Additionally, the impact of excipients on the dynamic binding capacity of the cation-exchange (CIEX) column was evaluated. **Results**: All tested excipients significantly reduced aggregation of both mAbs during low-pH incubation. For instance, mannitol reduced aggregation by sixfold in pembrolizumab and by threefold in denosumab. These additives enhanced conformational stability, as evidenced by the increased melting temperatures (Tm), with a pronounced stabilizing effect on the CH2 domain at low pH. Furthermore, the presence of sugars and polyols was associated with higher K_D_ values, indicating improved colloidal stability under acidic conditions. Importantly, adding these sugars or polyols did not impact the dynamic binding capacity of the CIEX column used in subsequent processing. **Conclusions**: This study offers valuable insights and supports the rational use of polyols and sugars for stabilizing mAbs during the low-pH VI step.

## 1. Introduction

Antibody-based therapeutics have increasingly gained dominance in the therapeutic market in the last decades [1,2]. These antibody-based therapeutics, such as monoclonal antibodies (mAbs) or fusion proteins, are commonly expressed in recombinant mammalian expression systems, which inherently pose the risk of viral contamination [3,4]. To ensure product safety, the purification process for these biotherapeutics incorporates at least two orthogonal steps specifically designed to remove and/or inactivate any potential viral threats [5]. Regulatory requirements mandate demonstrating the capacity of the purification [6,7] process for viral clearance. Established guidelines, such as the International Conference on Harmonization (ICH) Q5A, outline the requirements for evaluating and validating viral clearance in purification processes [8].

The primary techniques for virus removal are chromatographic separation and nanofiltration. To inactivate enveloped viruses, commonly used viral inactivation (VI) methods include exposure to low pH and/or treatment with solvent/detergent combinations. Conventionally, low-pH treatment (e.g., pH ≤ 3.6) of mAbs is routinely used to inactivate enveloped viruses due to the robustness of the method and ease of incorporation in downstream processes. Typically, the low-pH VI step is conveniently performed following Protein A capture chromatography since the mAb is eluted from the Protein A column using a mobile phase with pH ranging from 3 to 4. Following elution, the eluate is stirred and titrant is added to adjust the pH to around pH 3.5 and held for 30–60 min to ensure sufficient VI. The product stream then proceeds to further downstream processing by neutralizing it with an appropriate amount of base. This contrasts with the detergent/solvent VI treatment, which may require additional steps to ensure the complete removal of the detergent and solvent, as well as the potential generation of hazardous waste. However, the low-pH VI method is not without its problems. Proteins can undergo conformational changes and aggregation when exposed to acidic conditions. Additionally, some studies have also shown evidence of Protein A chromatography increasing the rate of aggregation during low-pH VI hold due to conformational changes of the proteins [6,9]. Consequently, low-pH exposure has been suggested as a stressor to examine antibody sensitivity to pH and their aggregation pathways, aiding in the rational design of manufacturing processes [10,11,12,13].

Among approved mAb-related therapeutics, most belong to the immunoglobulin (Ig) G class, with the IgG_1_ subclass being the most clinically used due to its potent effector function and excellent stability. IgG_4_ and IgG_2_ have also become important for therapeutics, requiring reduced or no effector function. Different IgG subclasses exhibit different conformational preferences and thermodynamic stability [14,15]. The propensity for aggregation among these subclasses has been ranked as follows: IgG_1_ < IgG_2_ < IgG_4_, over a broad pH range under thermal stress [14,16,17]. Similarly, in acidic conditions, IgG_1_ also exhibits the highest stability, while IgG_4_ shows the highest propensity to aggregate [10,18]. Consequently, native IgG_4_ molecules pose significant manufacturing challenges [18], and many protein engineering efforts have been made to stabilize IgG_4_ molecules [19]. Recently developed multi-specific antibody formats, such as bispecific antibodies and fusion proteins, are typically purified in a manner similar to standard mAbs. However, due to their structural complexities, they may be particularly sensitive to the extreme pH conditions often encountered during Protein A chromatography and viral inactivation [20]. This sensitivity can compromise protein quality and result in product loss, further complicating the purification and viral inactivation processes for these pH-sensitive antibodies. Therefore, strategies to address aggregation issues are necessary.

In response to this challenge, efforts have been made to modify the Protein A chromatography process, such as optimization of resin to allow milder pH elution [21] or the addition of additives in the elution buffer or in the eluate during low-pH VI treatment. These additives can include commonly used excipients, such as polyols, sugars, and amino acids, and also polymers like polyethylene glycols (PEGs) [6,18,22,23]. The beneficial effects of using these compounds to stabilize proteins are well documented in the literature. A common explanation is that polyols and sugars are preferentially excluded from the surface of the mAbs. This exclusion increases the protein-water chemical potential, thereby enhancing the thermodynamic stability of the protein against unfolding. As such, polyols and sugars are well regarded for their ability to stabilize protein conformation and protect them from the effects of stress conditions. Despite their widespread use, there has been limited research specifically examining the impact of polyols and sugars on the stability of mAbs during low-pH VI operations. The potential influence of these excipients on the efficiency of low-pH VI is also a critical consideration, as it can affect both product safety and downstream processing. Notably, a study by Stange et al. [22] demonstrated that sugars and polyols do not interfere with viral inactivation, supporting their use as effective agents for aggregation control during low-pH VI operations.

Since the IgG_1_ subclass of mAbs is generally stable under low-pH conditions, recent attention has shifted to the less stable IgG subclasses, such as IgG_4_ and IgG_2_, which are more prone to aggregation. Additionally, it has been reported that different IgG subclasses follow distinct aggregation pathways at acidic pH [7]. Therefore, understanding how additives influence the aggregation mechanisms of these subclasses during pH shifts in the VI process is crucial for developing a rational aggregation control strategy.

In this study, we aim to demonstrate that the addition of polyols and sugars in Protein A eluate is an effective strategy to mitigate protein aggregation during low-pH VI operations. To achieve this, we examined the effects of commonly used stabilizers—namely mannitol, sorbitol, trehalose, and sucrose—on the aggregation behavior of two mAbs during low-pH VI treatment. Pembrolizumab (PEM) and denosumab (DENO), representing the IgG_4_ and IgG_2_ subclasses, respectively, were selected for this study due to their significant aggregation observed during initial low-pH incubation screenings. The study was carefully designed to replicate the conditions encountered in the VI step during manufacturing. Previous research has shown that mAbs are more prone to aggregation after Protein A chromatography due to conformational changes, with aggregation typically starting after the subsequent neutralization step due to electrostatic repulsion in low-pH (or low ionic strength) conditions [6,24]. Therefore, the study included Protein A purification to mimic the conformational change during the actual process, and aggregation was monitored over a period of time after neutralization. To further understand the role of excipients on the stability of the mAbs, we critically examined conformational and colloidal stability at each processing condition for the two mAbs. The CH2 domain of the mAbs is notably destabilized at low pH; hence, we focused on the effects of excipients on the melting temperatures of the CH2 domain in the presence of different stabilizers. Intermolecular interactions often correlate with protein colloidal stability and mAb aggregation propensity. Consequently, we employed the diffusion interaction parameter (K_D_) and second virial coefficient (B_22_) to characterize the colloidal stability of mAbs with the various excipients under different pH conditions. Lastly, to assess the impact of excipients added during the VI step on subsequent purification steps, we examined their effect on the dynamic binding capacities (DBC) in cation-exchange chromatography. This study not only provides insight into the role of polyols and sugars in the aggregation behaviors of the mAbs under low-pH VI conditions but also presents a viable option for mitigating aggregation in downstream processing.

## 2. Results

### 2.1. Effect of pH on the Stability of Pembrolimumab (PEM) and Denosumab (DENO)

The impacts of low-pH treatment on the aggregation and protein recovery of PEM and DENO were studied across a pH range of 3.1 to 3.5, covering a wider range than typically applied for VI. Both PEM and DENO experienced extensive aggregation and protein loss as the pH decreased (Table 1). Notably, PEM aggregate levels rose considerably from 2% at pH 3.5 to over 40% at pH 3.2 after 1 h of incubation and neutralization. PEM exhibited greater pH instability with significantly higher aggregation and a greater loss of protein compared to DENO. While percent aggregate reflects soluble aggregates, protein recovery also accounts for losses due to precipitation. In severe cases, such as PEM at pH 3.1, both metrics are essential to accurately reflect the extent of degradation. For subsequent studies on the effect of additives on these two mAbs, a pH level where each mAb showed a propensity for aggregation was intentionally selected. Accordingly, pH 3.3 was chosen for PEM and pH 3.2 for DENO for subsequent low-pH treatment experiments.

### 2.2. Effect of Additives on the Stability of PEM During Low-pH VI Operation

To investigate representative aggregation behaviors of PEM under low-pH VI operation, PEM was subjected to a series of processing steps, including Protein A chromatography, low-pH incubation, and subsequent neutralization. Various additives, namely mannitol, sorbitol, trehalose, and sucrose, were evaluated for their effectiveness when added to the eluate. The impacts on PEM aggregation levels and charge variant profiles of PEM were then assessed following the low-pH incubation and neutralization.

#### Aggregation and Charge Variant Profiles

**Figure 1:**
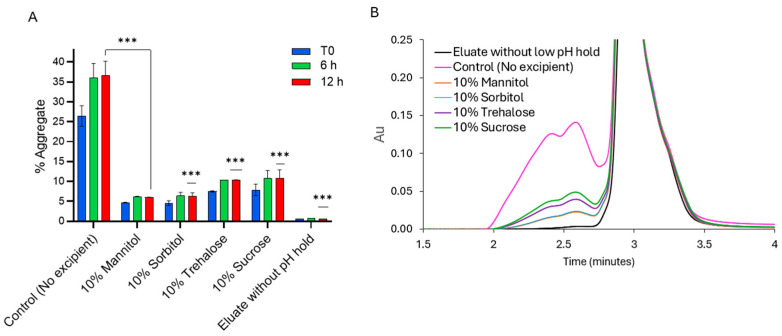
Aggregate levels (**A**,**B**) overlay of SEC chromatograms of PEM eluate with 10% *w*/*v* excipients following low-pH hold at pH 3.3 for 1 h and subsequent neutralization to pH 5.5. T0 denotes the aggregate level immediately after neutralization, while 6 h and 12 h denote aggregate levels at 6 and 12 h, respectively, upon standing after neutralization. The data presented is an average of duplicate samples. *** *p* < 0.001 using one-way ANOVA compared to Control (No excipient).

Ideally, changes in the aggregate and charge profiles of the mAb should remain minimal following exposure to VI processes, comparable to levels observed in eluate samples that have not undergone a low-pH hold. However, as shown in Figure 1, exposure of PEM to various VI processes led to extensive aggregation, reaching approximately 37%, in the control sample. In contrast, when 10% *w*/*v* of additives were included in the PEM eluate, aggregation was significantly reduced, ranging from 6% to 10%. All tested additives—mannitol, sorbitol, trehalose, and sucrose—proved highly effective in mitigating PEM aggregation, with polyols showing particularly strong effects. Additionally, aggregation was observed to increase progressively over 6 h. This observation aligns with previous findings indicating that, under acidic pH and low ionic strength, electrostatic repulsion prevents partially unfolded mAb molecules from aggregating, whereas neutralization reduces this repulsion and initiates aggregation [24]. The process of refolding can take several hours, leading to the formation of higher-order aggregates [23].

**Figure 2:**
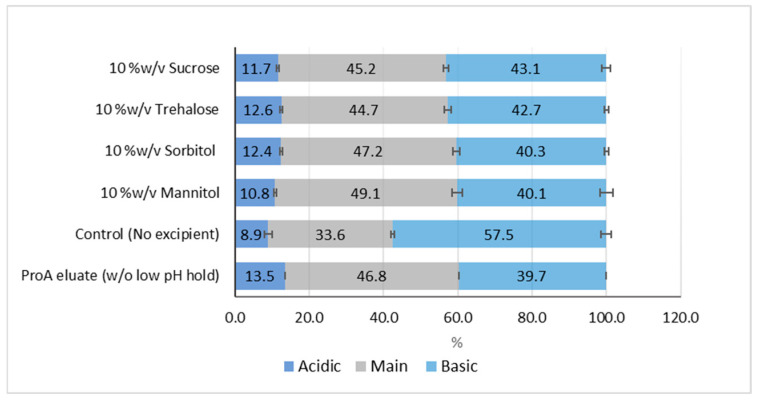
Changes in charge variant profile of PEM with and without low-pH hold in the presence of different excipients. The presented data is an average of duplicate samples.

To evaluate the charge profile of PEM after VI treatment, cation-exchange chromatography (CEX) on HPLC was employed. As shown in Figure 2, the control sample without additives exhibited a substantial increase of approximately 18% in basic variants, while the acidic variants decreased by 4.6%, compared to the PEM sample without low-pH exposure. These changes collectively led to a 13.2% reduction in the main species. In contrast, PEM eluates containing additives exhibited minimal changes in their charge variant profiles. Specifically, mannitol and sorbitol were more effective at preserving charge variant stability compared to trehalose and sucrose. This trend aligns with the aggregation patterns observed in Figure 1.

### 2.3. Conformational Changes in mAb Structure Following Protein a Chromatography and Low-pH Treatment

Nano differential scanning fluorimetry (nanoDSF) was used to assess the effects of Protein A chromatography, low-pH incubation, and the presence of excipients on the conformational stability of the protein.

#### 2.3.1. Effect of Protein a Chromatography and Low pH on the Melting Temperature of PEM

**Figure 3:**
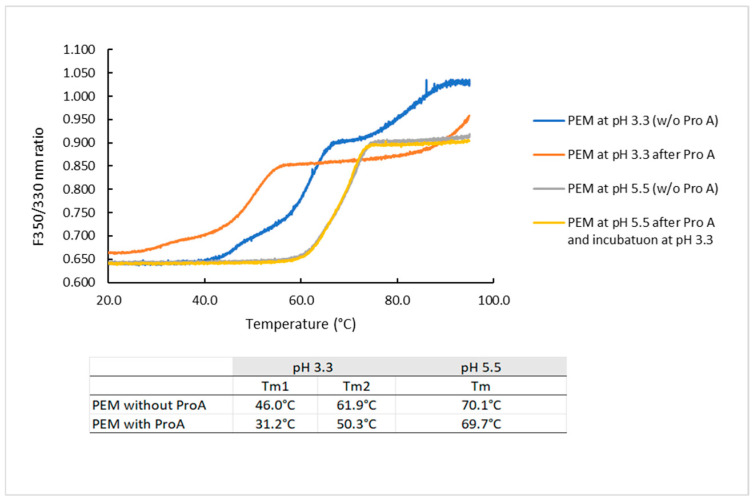
Effect of Protein A chromatography and low pH on the conformation and melting temperature of PEM analyzed by nanoDSF.

To study the impact of Protein A chromatography on the stability of PEM, the melting temperature (Tm) of a protein was used as an indicator of its thermal or conformational stability. The thermal unfolding curves of PEM subjected to Protein A chromatography were compared with those of PEM not subjected to this process (Figure 3). For the “PEM at pH 3.3 after Pro A” sample, the protein was first eluted from the Protein A column and then adjusted to pH 3.3, while for the “PEM pH 5.5 after Pro A” sample, eluate was first incubated at pH 3.3 prior to adjustment to pH 5.5. Samples that did not undergo Protein A chromatography were buffer exchanged directly to the respective pH values (i.e., 3.3 and 5.5). At pH 3.3, the PEM sample that underwent Protein A chromatography displayed a markedly altered unfolding profile compared to the one that did not (Figure 3). The unfolding transitions of PEM, as indicated by the inflection points (Tm1 and Tm2) of the unfolding curves, occurred at significantly lower temperatures in the Protein A chromatography-processed samples (Tm1: 31.2 °C vs. 46.0 °C; Tm2: 50.3 °C vs. 61.9 °C). These results confirm the destabilizing effect of Protein A chromatography on the IgG_4_ mAb. At pH 5.5, the unfolding transitions are less distinct, with only one prominent Tm observed. Interestingly, the unfolding curves overlapped and showed no effect from the prior Protein A chromatography step. The difference in Tm values was also minor compared to when the samples were at pH 3.3.

#### 2.3.2. Effect of Excipients on PEM Conformational Stability During Low-pH Treatment

**Figure 4:**
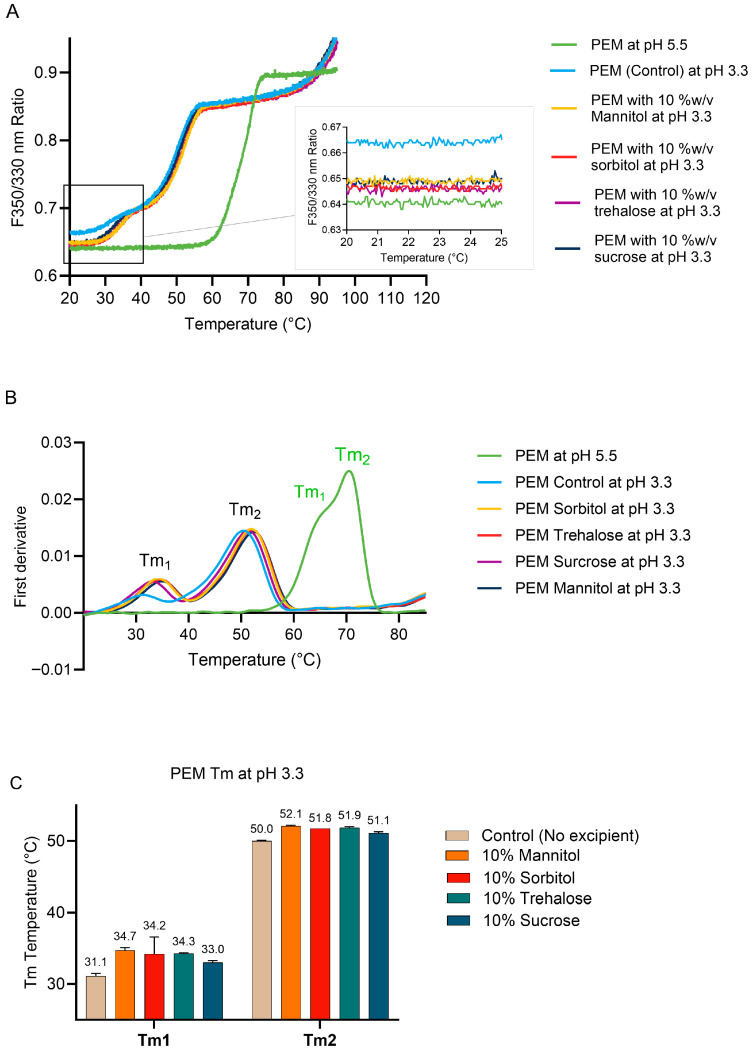
Effect of 10% *w*/*v* excipients on the (**A**) conformation of PEM at pH 3.3, (**B**) first derivative of unfolding profile of PEM during temperature melting from 20 to 85 °C, and (**C**) melting temperatures of PEM.

Thermal denaturation processes of PEM at pH 5.5 and pH 3.3 in the presence of different excipients are shown in Figure 4. The expanded section in Figure 4A compares the conformation of these PEM samples before thermal denaturation. A higher F350/330 ratio indicates more hydrophobic tryptophan residues of a protein are being partially exposed to the aqueous environment. Hence, it was observed that PEM at pH 5.5, being in its native form, has the lowest F350/330 ratio compared to PEM at pH 3.3. As previously observed in Figure 3, PEM at pH 3.3 exhibited a significant shift toward a higher F350/330 ratio (from 0.640 to 0.665). The addition of sugars and polyols, however, was able to reduce the F350/330 ratios to be closer to that of the native PEM compared to the PEM control without any excipient. Figure 4B shows the first derivative of the F350/330 ratios against temperature. The inflection points (Tm_1_ and Tm_2_) were clearly identified as the peaks in the graph. IgG antibodies generally exhibit multiple thermal transitions corresponding to the unfolding of their structural domains (e.g., CH2, CH3, Fab). For IgG4 monoclonal antibody, the first thermal transition (Tm1) is associated with the CH2 domains in the Fc region, while the Fab and CH3 were unresolved in the second unfolding transition (Tm2) [14,25,26]. It is evident that the CH2 domain is the least stable and highly sensitive to the pH of the solution. The presence of sugars and polyols resulted in shifts to higher Tm_1_ and Tm_2_ temperatures. As seen in Figure 4C, Tm_1_ (corresponding to the CH2 domain) of PEM increased by 1.9–3.6 °C in the presence of polyols or sugars. Among them, mannitol caused the highest increase inTm_1_. In contrast, the effect of these excipients on Tm_2_ (i.e., Fab region) was less pronounced, with increases ranging only from 1.1 to 2.1 °C.

### 2.4. Effect of Excipients on the Stability of DENO at Low pH

**Figure 5:**
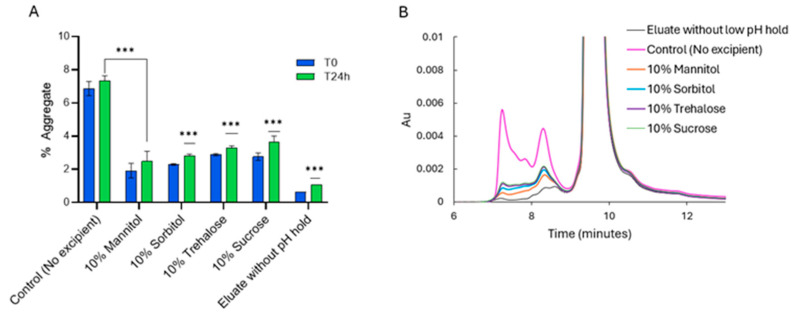
Aggregate levels (**A**,**B**) overlay of SEC chromatograms of DENO eluate with 10% *w*/*v* excipients after low-pH hold at pH 3.2 for 1 h and subsequent neutralization to pH 5.5. T0 denotes aggregate level immediately after neutralization, and T24 h denotes aggregate levels at 24 h, upon standing after neutralization. *** *p* < 0.001 using one-way ANOVA compared to Control (No excipient).

The study was extended to DENO, an IgG_2_ subclass monoclonal antibody, to determine whether polyols and sugars are also effective in suppressing acid-induced aggregation in this subclass. Similar to the procedure used for PEM, DENO was first subjected to Protein A chromatography, followed by low-pH incubation at pH 3.2 for 1 h and subsequently neutralized to pH 5.5. Figure 5A shows the aggregate levels of DENO eluate after the abovementioned steps. Unlike PEM, the control sample of DENO showed a moderate aggregate level of approximately 6–7%. However, the addition of 10% polyols or sugars significantly reduced aggregation, achieving a 2- to 3-fold decrease. As observed with PEM, polyols were generally more effective than sugars in reducing aggregation. Like in PEM, the formation of higher-order aggregates was gradual, and aggregate levels were monitored at 24 h post-neutralization until the growth of aggregate plateaued.

**Figure 6:**
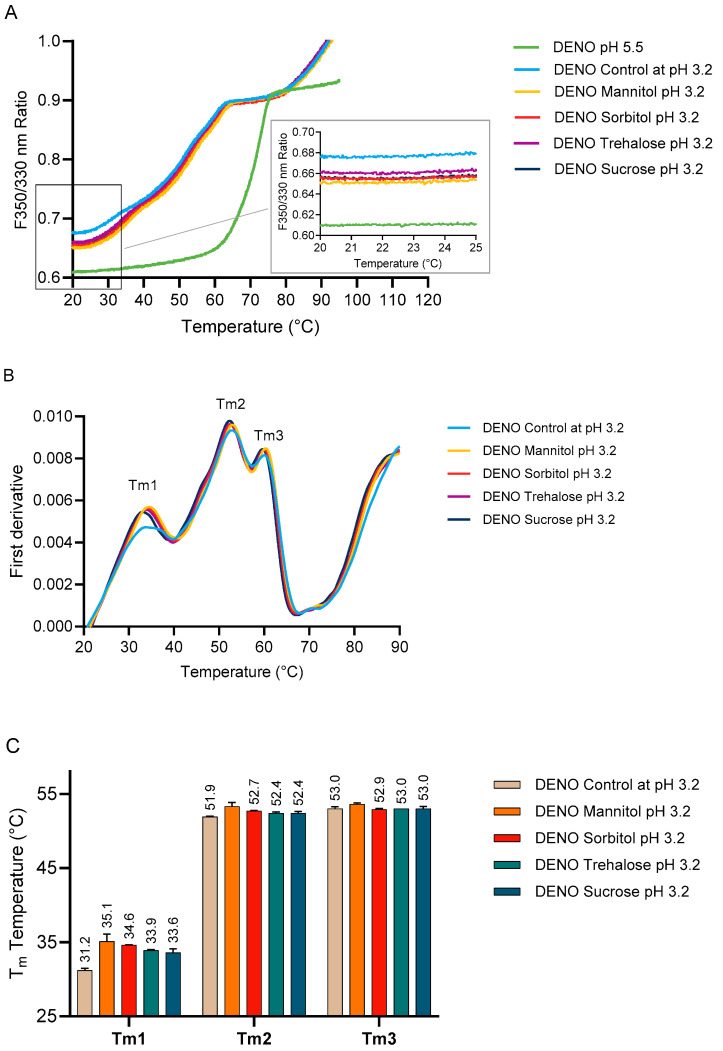
Effect of 10% *w*/*v* excipients on the (**A**) conformation of DENO at pH 3.2, (**B**) first derivative of unfolding profile of DENO during temperature melting from 20 to 85 °C, and (**C**) melting temperatures of DENO.

Although the effect of Protein A chromatography was not assessed in DENO, its conformational stability in the presence of polyols and sugars was assessed using nanoDSF. Figure 6A shows the full unfolding profile of DENO under different pH conditions and in the presence of different excipients. As observed previously, pH has a significant impact on the monoclonal antibody’s conformation—DENO at pH 5.5 and pH 3.2 displayed markedly different unfolding behaviors. The expanded section in Figure 6A compares the conformation state of DENO before thermal denaturation. At pH 3.3, DENO exhibited a much higher F350/330 ratio compared to pH 5.5 (0.68 vs. 0.61), indicating a compromised conformational state. However, in the presence of polyols and sugars, the F350/330 ratios decreased to approximately 0.65–0.66, suggesting a state closer to the conformation state at pH 5.5. Figure 6B shows the first derivative of the F350/330 ratios plotted against temperature. For IgG_2_ mAb, the inflection points, i.e., Tm_1_, Tm_2,_ and Tm_3,_ corresponded to the CH2 domain, Fab, and CH3 domain, respectively [14,25,26]. The addition of polyols and sugars caused the highest change in Tm_1_ (corresponding to the CH2 domain), leading to an increase of Tm by 2.4–3.9 °C. In contrast, changes in Tm_2_ and Tm_3_ were minimal.

### 2.5. Effect of Excipients on Colloidal Stability at Low pH and Post-Neutralization Conditions

**Figure 7:**
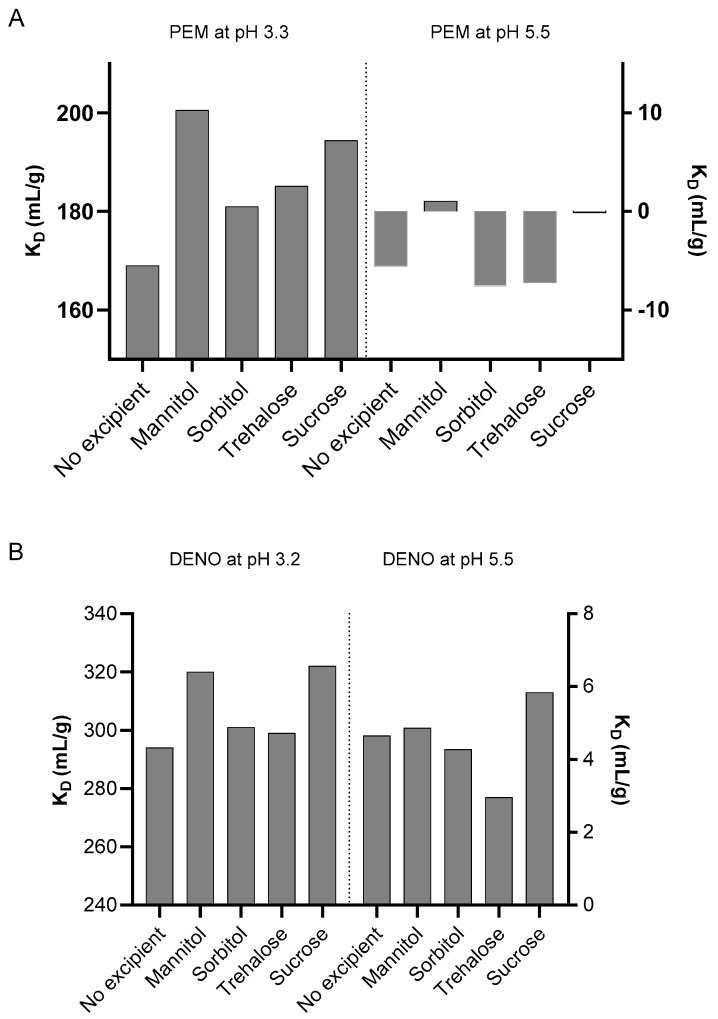
Effect of 10% *w*/*v* excipients on the K_D_ of (**A**) PEM at pH 3.3 and pH 5.5, (**B**) DENO at pH 3.2 and pH 5.5. Dashed lines represent division of graphs into two different pH regions.

The diffusion interaction parameter (K_D_) and the second virial coefficient (B_22_) are widely recognized for assessing protein colloidal stability and aggregation propensity [27,28]. To assess the impact of excipients on the colloidal stability of mAbs during low-pH VI processes, the parameters K_D_ and B_22_ were measured using dynamic light scattering (DLS) and static light scattering (SLS). Figure 7 shows the K_D_ values for PEM and DENO at pH 3.3 and pH 5.5 in the presence of different excipients. Corresponding B_22_ data can be found in Supplementary Figure S1. At pH 3.3, both PEM and DENO exhibited strongly positive K_D_ values, indicating high colloidal stability. This observation corroborated our previous observations that mAb aggregation did not occur during low-pH incubation, likely due to strong intermolecular repulsions. Instead, association or aggregation of partially unfolded monomers seemed to occur post-neutralization at pH 5.5. This is further supported by the negative K_D_ value observed for PEM and the slightly positive K_D_ value for DENO at pH 5.5. In the presence of polyols and sugars, K_D_ values at pH 3.3 further increased, suggesting that these excipients enhance colloidal stability at low pH. However, at pH 5.5, the effects of polyols and sugars on K_D_ values are less definitive. In some cases, sorbitol and trehalose appeared to decrease K_D_ values slightly, while mannitol and sucrose appeared to increase K_D_ values modestly for both PEM and DENO.

### 2.6. Effect of Polyols and Sugars on the Dynamic Binding Capacity of CIEX Column

**Figure 8:**
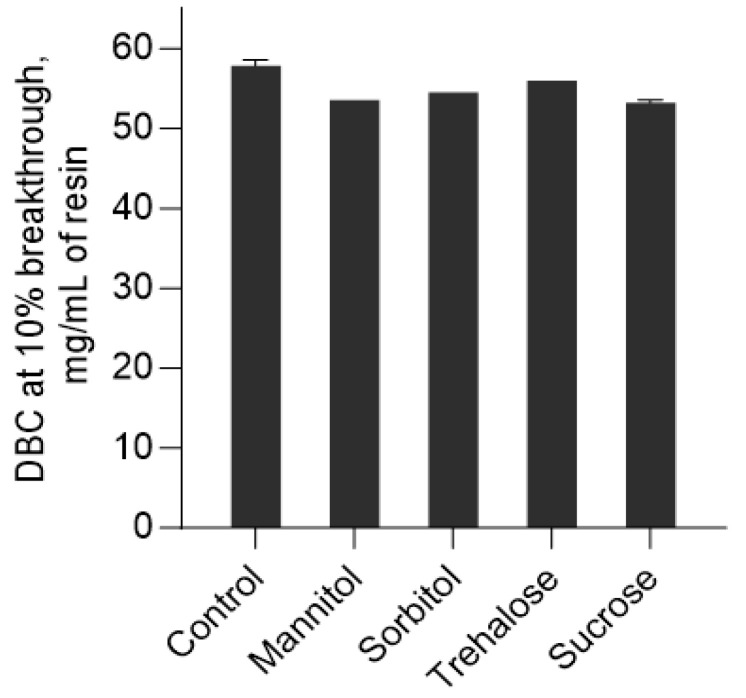
Dynamic binding capacity of CIEX column at 10% breakthrough in the absence and presence of polyols and sugars.

To assess whether excipients added during low-pH viral inactivation affect subsequent chromatography steps, the dynamic binding capacity (DBC) of a cation-exchange (CIEX) resin—representing a potential next purification step—was evaluated using denosumab samples. At a residence time of 1 min, approximately 55–60 mg of denosumab per mL of resin could be bound before reaching 10% breakthrough in the absence of any excipient (Figure 8). The inclusion of polyols or sugars led to a slight reduction in DBC, with binding capacities of approximately 53–56 mg/mL. However, the differences observed among the tested excipients—mannitol, sorbitol, sucrose, and trehalose—were not significant.

## 3. Discussion

Low-pH conditions are frequently encountered during the production of mAbs and other therapeutic proteins, particularly during processes like Protein A chromatography and virus inactivation (VI) steps. Acid-induced aggregation of mAbs has not been a significant concern for the widely used IgG_1_ mAbs, given their remarkable stability at low pH. However, acid-induced aggregation of IgG_2_ and IgG_4_ subclasses has been well documented and necessitates an aggregation control strategy. In our study, PEM (IgG_4_) experienced much higher levels of aggregation than DENO (IgG_2_) at low pH. This observation is consistent with previous findings that IgG_4_ exhibits the highest propensity for aggregation under acidic conditions compared to other IgG subclasses [13,15,17], a behavior attributed to hydrophobic motifs in the Fc region of IgG_4_ monoclonal antibodies [28]. The results also underscore the importance of understanding the aggregation behavior of proteins during the virus inactivation-associated operations and developing strategies to mitigate aggregation.

Sugars and polyols are an important class of excipients commonly included in therapeutic protein formulations to improve stability during storage. Besides being known to reduce thermal-induced aggregation by enhancing conformational stability [29], sugars and polyols have also been reported in several studies to reduce acid-induced aggregation [7,18,22,25]. As expected, all studied excipients—mannitol, sorbitol, trehalose, and sucrose—showed effectiveness in controlling protein aggregation, as well as changes in charge profile, when added at 10% *w*/*v* to the Protein A eluate (Figure 1, Figure 2 and Figure 5). However, it is noteworthy to point out that sucrose might not be the most suitable stabilizer under acidic conditions. Sucrose can undergo acid hydrolysis, leading to glycation in protein therapeutics [30,31].

In addition to their role in preventing aggregation, sugars and polyols also influence the charge variant profile of proteins, which can reflect differences in net charge, as well as changes in the distribution of charges on the protein surface [32]. The large increase in basic variants in PEM following low-pH incubation can be due to chemical modifications such as deamidation and oxidation [33,34], changes in protein conformation [35], or a combination of both. Notably, the addition of polyols and sugars in PEM eluates was found to minimize changes in charge heterogeneity, indicating the effectiveness of these excipients to resist either chemical modifications or conformational changes.

While numerous studies have evaluated excipients as a strategy to mitigate aggregation under acidic conditions, few have investigated their impact on the conformational and colloidal stability of mAbs during low-pH VI processes. Conventionally, low-pH VI is performed immediately following the Protein A chromatography step, as the mAb is typically eluted from the Protein A column using an acidic mobile phase. This acid elution induces transient conformational changes, increasing mAb susceptibility to aggregation [36], with aggregation rates reported higher than from low-pH exposure alone [7,25]. To evaluate conformational stability under these conditions, we assessed the impact of Protein A chromatography and low-pH incubation on mAb conformational stability using nanoDSF, which monitors conformational changes through changes in intrinsic fluorescence and determines melting temperature (Tm) [37,38]. The fluorescence intensity ratio at 350 nm to 330 nm (F350/330) reflects changes in the polarity of the environment around tryptophan residues. As tryptophan side chains become more exposed to a polar, aqueous environment (e.g., during protein unfolding), the emission maximum typically shifts from 330 nm to 350 nm, resulting in an increased F350/330 ratio [39].

Remarkably, the thermal unfolding curve of PEM following Protein A chromatography and incubation at pH 3.3 was distinctly altered (Figure 2), indicating a substantial loss of conformational or thermal stability. The results highlighted the susceptibility of PEM, an IgG_4_ mAb, to the combined effects of Protein A chromatography and low-pH exposure. Interestingly, at pH 5.5, the unfolding transition appeared to be unaffected by prior Protein A chromatography and low-pH incubation, suggesting effective structural recovery following the initial conformational change. A similar observation was also reported by Mazzer et. al, where the recovered (re-folded) monomer was not found to be distinct from the initial (unfolded/native) monomer [6].

At low pH, both PEM and DENO control samples exhibited higher F350/330 ratios compared to those at pH 5.5 (Figure 4 and Figure 6), suggesting increased exposure of tryptophan residues to a polar environment, likely due to conformational changes. The addition of sugars and polyols reduced the F350/330 ratios in both PEM and DENO eluates. It should be noted that sugars and polyols may also alter solvent polarity, thereby affecting the tryptophan fluorescence spectrum. The stabilizing effect of these excipients was further supported by the observed increase in the Tm values of the mAbs. For both PEM and DENO, the first thermal transition (Tm_1_) corresponds to the unfolding of the CH2 domains, which is known to be the least thermally stable region of the IgG molecule under both neutral and acidic conditions [17]. The addition of sugars and polyols led to a greater increase in Tm_1_ (CH2 domain) than in Tm_2_ (Fab region). Notably, mannitol and sorbitol caused a higher increase in Tm_1_ values compared to trehalose and sucrose. This higher Tm increase is loosely correlated to the lower levels of aggregation observed. Overall, these results suggest that under acidic conditions, the CH2 domains of PEM and DENO are partially unfolded, leading to exposure of hydrophobic regions of the Fc region. The presence of these tested polyols or sugars likely shielded the hydrophobic patches of the CH2 domain, thereby enhancing the thermal stability of the CH2 domain and reducing aggregation in PEM and DENO. These results align with other studies whereby the presence of D-sorbitol, mannose, and sucrose was found to lower ANS binding of mAb solutions at low pH [24,25]. Overall, our findings provide critical insights into the use of polyols and sugars for managing the aggregation of IgG_4_ and IgG_2_ subclass mAbs during low-pH virus inactivation processes.

Colloidal stability of mAbs during the low-pH virus inactivation (VI) process is often overlooked in many studies. While some studies assess aggregation under low pH and high ionic strength, actual processing typically involves low pH and low ionic strength, as mAbs are eluted with a low ionic strength acidic mobile phase before acidification. Under these conditions, Wälchli et al. reported that the high protein surface charge, coupled with limited electrostatic screening due to the low ion concentration, prevents aggregation through electrostatic repulsion between mAb molecules [24]. Our strongly positive K_D_ and B_22_ values (Figure 7 and Figure S1) also indicate strong repulsive forces between the mAb molecules and high colloidal stability. This suggests that, despite the compromised conformational state of mAbs at pH 3.3 (based on nanoDSF analysis), the strong repulsive forces at low pH and ionic strength prevented oligomerization. Oligomerization appears to occur primarily during the neutralization phase, when an increase in pH and ionic strength reduces repulsive force, enabling self-association of the partially denatured monomers. This is evidenced by the significant drop in K_D_ values at pH 5.5 for both PEM and DENO. Thus, the neutralization process can be visualized as a sudden reduction in electrostatic repulsion caused by the increased pH and ionic strength. During the phase, a kinetic competition between the refolding of the denatured molecules and their self-association is presumably taking place [6,24]. The role of polyols and sugars in this kinetic competition is particularly intriguing. We observed more positive K_D_ values in the presence of polyols and sugars at pH 3.3 for both PEM and DENO, indicating enhanced conformational stability and colloidal stability by polyols and sugars. However, this stabilizing effect was not observed for all polyols and sugars at pH 5.5. Only mannitol and sucrose yielded improved K_D_ values compared to the control. This observation suggests that the colloidal stability at pH 5.5 does not directly correlate with the aggregation level of mAbs following low-pH viral inactivation treatment. Instead, the extent of aggregation is influenced more by the antibody’s colloidal stability during the low-pH incubation phase rather than after neutralization.

Taking all these observations into account, we propose the following stabilization mechanism for polyols and sugars during low-pH VI treatment:

1. Low pH causes partial or complete unfolding of mAbs. This effect is further exacerbated by prior Protein A chromatography, which increases the likelihood of structure destabilization.
2. During low-pH incubation, mAbs remain in a partially unfolded state but typically do not aggregate due to strong electrostatic repulsion. The addition of polyols or sugars enhances the conformational stability of mAbs by reducing the exposure of hydrophobic residues. This enhances colloidal stability by reducing attractive forces between mAb molecules.
3. Upon neutralization, with improved conformational and colloidal stability in the presence of polyols and sugars at low pH, the conformational equilibrium of the mAbs is shifted more toward the native state rather than the partially unfolded state. As a result, partially unfolded mAbs are more likely to refold correctly, rather than self-associate, thereby reducing aggregation.

Finally, beyond their effectiveness, sugars and polyols are practical stabilizing excipients for reducing aggregation during low-pH VI. Importantly, the addition of sugars or polyols to the Protein A eluate did not compromise subsequent downstream processes. Our results demonstrated that sugars and polyols did not affect the dynamic binding capacity of CIEX columns. Furthermore, Stange et al. also showed that virus inactivation was not impacted in the presence of these additives [22]. This study has demonstrated that these compendia excipients can effectively stabilize proteins during low-pH conditions, without affecting efficiency of virus inactivation or subsequent downstream processing steps.

## 4. Materials and Methods

### 4.1. Materials

Pembrolizumab (PEM) was purchased from BOC Sciences (Shirley, NY, USA), and denosumab (DENO) was produced in-house from a licensed CHO cell-line (CHO K1). Biopharma grade mannitol (PEARLITOL^®^) and sorbitol (NEOSORB^®^) were from Roquette Frères (Lestrem, France). α, α- trehalose dihydrate and sucrose were purchased from Merck KGaA (Darmstadt, Germany). Acetic acid, sodium phosphate (monobasic, monohydrate), and sodium phosphate dibasic, anhydrous) were purchased from Merck KGaA (Darmstadt, Germany). All other chemicals and reagents were used as received without further purification.

### 4.2. Methods

#### 4.2.1. Antibody Batch Protein a Purification, Low-pH Incubation and Neutralization

PEM and DENO were purified by batch Protein A purification using MabSelect PrismA^TM^ (Cytiva, Marlborough, MA, USA) resin. Briefly, proteins were incubated with a 50% slurry of Protein A resin in binding buffer (150 mM sodium phosphate, 150 mM sodium chloride, pH 6.8) for 1 h under gentle stirring. The protein-resin mixture was then loaded onto an open column and washed with binding buffer. Bound proteins were eluted with four column volumes (i.e., resin volume) of 50 mM acetic acid. All fractions of eluates were collected, and protein concentrations were determined by measuring absorbance at 280 nm using a NanoDrop UV spectrophotometer (Thermo Fisher Scientific, Waltham, MA, USA). Extinction coefficients (E1%) of 14.2 for PEM and 14.0 for DENO were used for the calculations. The resulting protein concentrations ranged from 4 to 5 mg/mL. The eluates were mixed and split equally. Eluates denoted as “T0” were immediately pH-adjusted to pH 5.5. Aside from the eluate denoted as “control”, excipients (i.e., mannitol, sorbitol, trehalose, and sucrose) were respectively added into the remaining Protein A eluates to afford a concentration of 10% *w*/*v*. Eluates were then individually adjusted to pH 3.3 for PEM or pH 3.2 for DENO using 2 M acetic acid. All samples were prepared in duplicates. All eluates were then incubated at the respective pH for 1 h before neutralization to pH 5.5 using 1 M Tris buffer, pH 9.0. Sample pH was always within ±0.02 units of the target value as independently verified by a calibrated Mettler Toledo Seven Compact S220 pH meter (Columbus, OH, USA). After neutralization, Size Exclusion Chromatography (SEC-HPLC) analyses were performed. The aggregate profile of the mab after neutralization was monitored immediately and also for a period of 4 to 24 h.

#### 4.2.2. Aggregate and Protein Recovery Determination by Size Exclusion Chromatography (SEC)-HPLC

Eluate samples were centrifuged at 21,000× *g* for 15 min at 4 °C to remove debris before HPLC analysis. For SEC-HPLC: Eluate samples were evaluated using a Waters Acquity HPLC with a XBridge BEH SEC column, 200 Å, 3.5 µm, 7.8 mm × 300 mm (Waters Corporation Milford, MA, USA). 10 µL of each sample was injected and eluted isocratically at a flow rate of 0.5 mL/min. For SEC-UPLC: Eluate samples were evaluated using a Waters Acquity UPLC with an ACQUITY Premier Protein SEC Column, 250 Å, 1.7 µm, 4.6 mm × 150 mm (Waters Corporation Milford, MA, USA). 10 µL of each sample was injected and eluted isocratically at a flow rate of 0.4 mL/min. Mobile phase of 25 mM sodium phosphate and 150 mM sodium chloride, pH 6.8, was used. Data was analyzed using Waters Empower 3 chromatography data system software (Waters Corporation, Milford, MA, USA, version 3). The relative amounts of monomers, aggregates, and fragments were determined by peak area integration. The percentage of aggregates was calculated as the ratio of the aggregate peak area to the total peak area. Protein recovery was determined by dividing the total peak area of the sample (adjusted for dilution resulting from pH adjustments) by the total peak area of the T0 sample.

#### 4.2.3. Cation-Exchange Chromatography (CEX) for Charge Heterogeneity Profiling of Pembrolizumab

A CEX-HPLC system with a Waters Protein-Pak^TM^ Hi Res WCX column (CM 7 μm, 4.6 × 100 mm) was used to analyze the charge variants of PEM. Using a fixed-pH (pH 6.1 with 25 mM of sodium phosphate) with a 0–150 mM sodium chloride gradient in 40 min, the CEX chromatographic separation of PEM was achieved. Acidic variants, main isoform, and basic variants were separated and analyzed to characterize the charge variant profiles.

#### 4.2.4. Buffer Exchange of Proteins to Different pH Conditions

To investigate the colloidal and conformational stability of PEM and DENO under varying pH conditions, proteins were buffer exchanged into 50 mM acetate buffer at either pH 3.3 or pH 5.5 using PD-10 Sephadex G-25M desalting columns (Cytiva, Marlborough, MA, USA). Specifically, 2.5 mL of protein solution was loaded onto each column and allowed to fully enter the packed bed. The proteins were subsequently eluted with 3.5 mL of the corresponding buffer.

#### 4.2.5. Nano Differential Scanning Fluorimetry (NanoDSF) Analysis and Tm Determination

NanoDSF was performed using Prometheus NT.48 equipped with backreflection mode (NanoTemper Technologies, Munich, Germany). Samples were loaded in nanoDSF-grade standard capillaries and subjected to thermal ramp from 20 °C to 95 °C at a rate of 1 °C/min. Thermal stability parameters, including the F350/330 nm ratio, were measured, and Tm, were calculated by PR. ThermControl software, version 2.1.2.

#### 4.2.6. Determination of K_D_ and B_22_ Values from Dynamic and Static Light Scattering

Dynamic and static light scattering measurements were made using Stunner (Unchained Labs, Pleasanton, CA, USA). The model mAbs (i.e., PEM and DENO) were first buffer exchanged into 50 mM acetate buffer, pH 3.3 or pH 5.5 using PD-10 Sephadex G-25M desalting column (Cytivia, Marlborough, MA, USA), and then concentrated to approximately 50 mg/mL using Amicon^®^ Ultra-15 Centrifugal Filter Units with a M.W. cut-off of 30 kDa (Merck Millipore, Burlington, MA, USA) by centrifugation at 4000× *g*. For each mAb, the concentrated mAb was diluted to 1, 2, 5, and 10 mg/mL, containing either 0% (control) or 10% *w*/*v* excipient (i.e., mannitol, sorbitol, trehalose, and sucrose) in the respective buffer. For each formulation, 2 µL of each sample was loaded in triplicate on a Stunner plate. The B_22_ and K_D_ application was selected using 4 DLS acquisitions of 5 s each, with water alone serving as blanks for each dilution series. Stunner Analysis software v7.0 was used to calculate K_D_ and B_22_ values. For K_D_, the diffusion coefficient was plotted as a function of the measured protein concentration at 280 nm. The slope and intercept of the plots were then used to calculate K_D_ values. For B_22_, the average scattering intensity of the measured light scattering intensity from each sample was used to calculate the Rayleigh ratio (R_θ_) value for each protein concentration. The linear fits of R_θ_ plotted against the measured protein concentration at 280 nm was then used to calculate B_22_ values. As K_D_ and B_22_ values are derived from the slope of their respective linear fits, a single value for each sample series was obtained.

#### 4.2.7. Dynamic Binding Capacity Measurements

Dynamic binding capacity (DBC) of cation-exchange resin for low-pH-treated samples was performed. Denosumab at a concentration of 4 mg/mL, added with different excipients (sugars or polyols), was injected into a HiTrap SP FF 1 mL column. The protein was applied at a flow rate of 1 mL/min until 10% breakthrough was reached. Excess unbound protein was washed off the column with equilibration buffer (50 mM sodium acetate, pH 5.5), and elution was achieved using a salt gradient to 1 M sodium chloride in 10 column volumes.

## 5. Conclusions

Most platform purification processes for antibodies and Fc-fusion proteins involve Protein A chromatography followed by low-pH VI operations. However, the recent development of new antibody formats and the increase in less pH-stable mAb subclasses, such as IgG_4_ and IgG_2_, have necessitated strategies to address aggregation issues caused by low-pH exposure during VI operations. Polyols and sugars, as tested in this study, have demonstrated effectiveness in mitigating aggregation in PEM and DENO after low-pH incubation. Although the stabilizing effects of polyols and sugars are well-documented in protein formulations, there is limited understanding of how these stabilizers influence the underlying aggregation mechanisms during low-pH VI operations. By employing nanoDSF and DLS analyses, we were able to deduce the conformational and colloidal states of the mAbs during acidic conditions. This work has taken a closer look at the role these polyols and sugars played during low-pH VI operation. It may also aid in providing a strong scientific rationale in the selection of suitable stabilizer(s) for other antibody formats. Lastly, this work has put forth a straightforward aggregation control strategy, using compendia stabilizers that can be seamlessly integrated into the product stream without causing any process incompatibility issues downstream or necessitating additional removal steps.

## Figures and Tables

**Table 1:** Percentage of aggregates and protein recovery for PEM and DENO after 1 h incubation at different pH values, followed by neutralization to pH 5.5. “T0” refers to the Protein A eluate that was immediately neutralized to pH 5.5, and protein recovery for all other pH conditions was normalized to this value. SD = standard deviation.

	PEM		DENO	
	Aggregate (%)	Protein Recovery (%)	Aggregate (%)	Protein Recovery (%)
	Mean (SD)	Mean (SD)		
T0	0.36 (0.01)	100.0	1.77	100.0
pH 3.5	2.05 (0.00)	99.6 (0.00)	2.63	99.5
pH 3.4	12.10 (0.02)	93.7 (0.1)	3.59	98.4
pH 3.3	36.05 (0.05)	85.2 (0.2)	5.82	98.7
pH 3.2	40.94 (0.21)	77.0 (0.9)	11.51	96.0
pH 3.1	35.68 (0.37)	69.2 (2.5)	30.54	88.1

## Data Availability

The original contributions presented in this study are included in the article/Supplementary Material. Further inquiries can be directed to the corresponding author.

## Supplementary Materials

The following supporting information can be downloaded at https://www.mdpi.com/article/10.3390/ph18121846/s1, Figure S1: Effect of 10% *w*/*v* excipients on the B22 of (A) PEM and (B)DENO at pH 3.3 and pH 5.5.
