# Supplementary figures and images for: Understanding the Role of Polyols and Sugars in Reducing Aggregation in IgG2 and IgG4 Monoclonal Antibodies During Low-pH Viral Inactivation Step
Source: Pharmaceuticals (Basel). 2025 Dec 3;18(12):1846. doi: 10.3390/ph18121846 (PMC12736099; doi:10.3390/ph18121846)

# ROQUETTE RESTRICTED

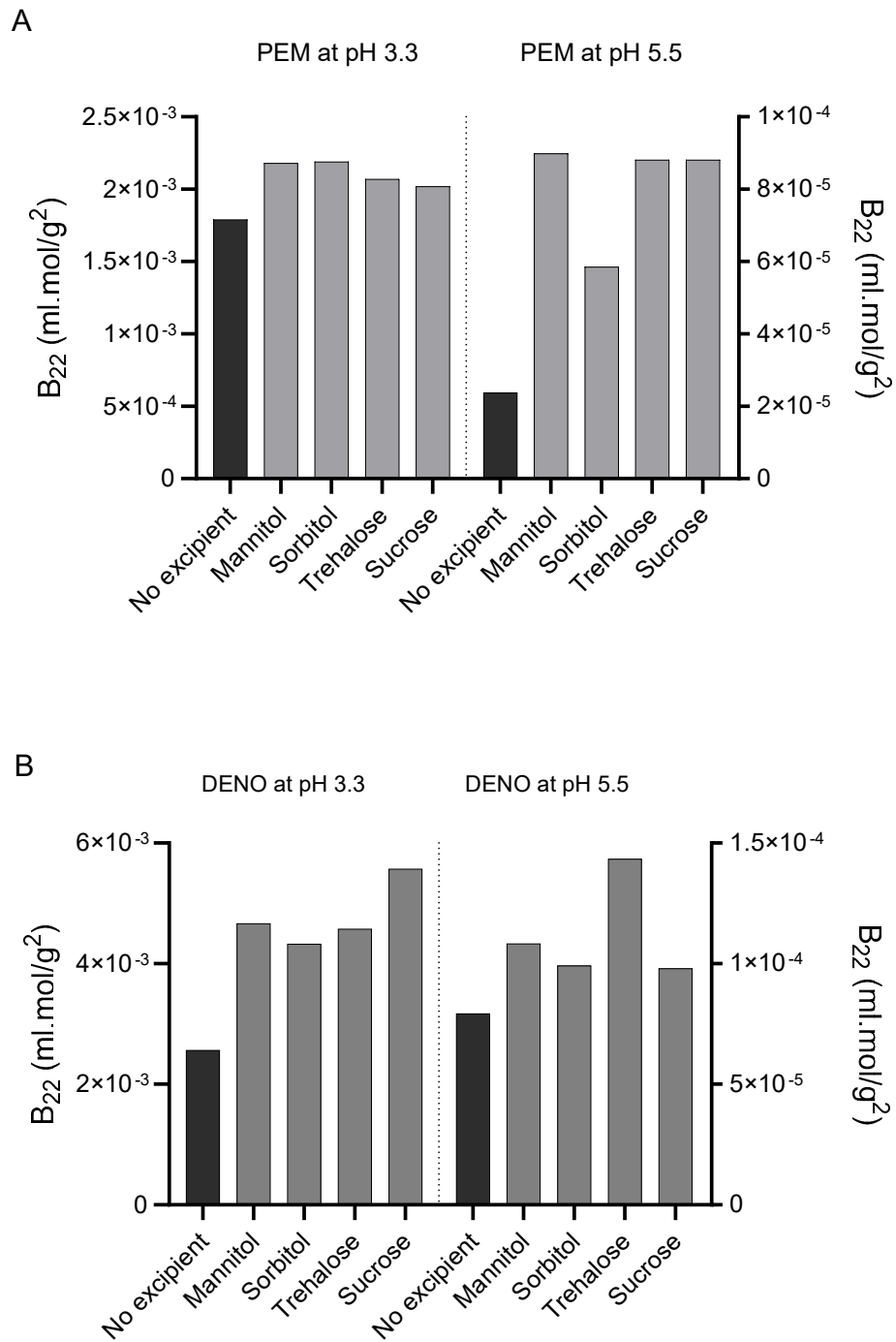

**Figure S1.** Effect of 10% w/v excipients on the  $B_{22}$  of (A) PEM and (B) DENO at pH 3.3 and pH 5.5.

Supplement: Supplementary file 1 [file pharmaceuticals-18-01846-s001.zip › pharmaceuticals-3982454-supplementary.pdf]
